# Manufacturing and Characterization of Femtosecond Laser-Inscribed Bragg Grating in Polymer Waveguide Operation in an IR-A Wavelength Range

**DOI:** 10.3390/s20010249

**Published:** 2020-01-01

**Authors:** Jan Meyer, Antonio Nedjalkov, Christian Kelb, Gion Joel Strobel, Leonhard Ganzer, Wolfgang Schade

**Affiliations:** 1 Department for Fiber Optical Sensor Systems, Fraunhofer Heinrich Hertz Institute, Am Stollen 19H, 38640 Goslar, Germany; antonio.nedjalkov@hhi.fraunhofer.de (A.N.); christian.kelb@hhi.fraunhofer.de (C.K.); wolfgang.schade@hhi.fraunhofer.de (W.S.); 2 EST Research Center Energy Storage Technologies, Clausthal University of Technology, Am Stollen 19A, 38640 Goslar, Germany; gion.joel.strobel@tu-clausthal.de (G.J.S.); leonhard.ganzer@tu-clausthal.de (L.G.)

**Keywords:** optical sensor, Bragg grating, laser direct lithography, femtosecond laser, photoconductive polymer, polymer waveguide

## Abstract

Optical sensors, such as fiber Bragg gratings, offer advantages compared to other sensors in many technological fields due to their outstanding characteristics. This sensor technology is currently transferred to polymer waveguides that provide the potential for cost-effective, easy, and flexible manufacturing of planar structures. While sensor production itself, in the majority of cases, is performed by means of phase mask technique, which is limited in terms of its degrees of freedom, other inscription techniques enable the manufacture of more adaptable sensor elements for a wider range of applications. In this article, we demonstrate the point-by-point femtosecond laser direct inscription method for the processing of polymer Bragg gratings into waveguides of the epoxy-based negative photoresist material EpoCore for a wavelength range around 850 nm. By characterizing the obtained grating back-reflection of the produced sensing element, we determined the sensitivity for the state variables temperature, humidity, and strain to be 45 pm/K, 19 pm/%, and 0.26 pm/µε, respectively. Individual and more complex grating structures can be developed from this information, thus opening new fields of utilization.

## 1. Introduction

Since the first discovery of fiber photosensitivity in the late seventies, fiber Bragg gratings (FBG) have been extensively studied and their outstanding performance for many different purposes are of high interest to this day [1]. In lightwave communications, these periodic refractive index modulations within a waveguide core are used as narrowband reflectors (e.g., for fiber lasers or laser wavelength stabilization), highly reflective mirrors (e.g., pump reflector in fiber amplifiers), or gratings (e.g., dispersion compensation for long-haul transmission) [1,2]. Besides these implementations, FBG are regarded as excellent sensor elements because their measurand information is wavelength-encoded, and therefore intrinsically immune to disturbances, such as power loss, and enable the measurement of the physical variables temperature and strain [1,3,4]. Furthermore, additional parameters such as pressure, humidity, chemical substances, and others can be measured indirectly with appropriate transducers. Moreover, FBG offer many advantages, as they are lightweight, flexible, and show high temperature tolerance and immunity to electromagnetic disturbance.

While the first demonstrated examples of Bragg sensors were inscribed in silica-based glass fibers, polymer materials are also known to be suitable for photonic components due to their low cost, easy manufacturing, simple control of material properties, and ability to produce flexible planar structures [5,6,7,8,9]. Among others, polymethyl-methacrylate (PMMA) is one example for a widely used polymer material, which offers sufficiently low optical loss for light transmission in optical telecommunication or sensor networks that are typically fiber-based. In recent years, however, many other polymeric materials have surfaced that offer beneficial optical properties and enable easy manufacturing with techniques such as laser direct lithography to produce microphotonic systems [10,11,12]. EpoCore, for instance, is a negative epoxy-based photoresist, designed to fabricate waveguides that have long-term stable material properties after processing [13].

In contrast to glass fiber gratings, Bragg sensors in polymer optical materials are usually more sensitive to strain and temperature. The increase in strain sensitivity is reported to be around 15%–22% in contrast to silica-based sensors because of the lower photoelastic constant. This feature can additionally be maintained over a broader range of applied strain due to the material’s high yield strain, although nonlinear effects have to be considered for larger distortions. For the temperature sensitivity of polymer-based Bragg gratings, not only are larger phase shifts (up to 800% increase in sensitivity) reported, but also with opposite signs than for silica-based sensors [5,6]. Because of a negative thermo-optic coefficient for most polymer materials, a decrease in the reflected Bragg wavelength with increasing temperature occurs. This is of particular interest for some applications, since it allows new sensor designs with intrinsic temperature compensation.

Historically, most Bragg sensors are reported at a wavelength of around 1500 nm, which is basically due to low absorption of glass materials in this range, the general telecommunications background of this technology, and also the increased sensitivity of Bragg sensors at higher wavelengths, as will be shown in Section 2.3 [1,2]. Planar polymer-based Bragg sensors, in principle, allow a flexible design of the waveguide and optical properties of the material [14]. Because a trend of further miniaturization and cost-optimization is expected, the use of Bragg sensors at a wavelength of around 800 nm is becoming increasingly interesting. This wavelength tolerates smaller waveguide dimensions in the case of single-mode applications, along with the use of silicon detectors.

For the inscribing of Bragg gratings in waveguides, regardless of the utilized material and shape, a variety of processes exist [1,2]. One major process is the phase mask technique, which uses diffractive optical elements (e.g., one-dimensional periodic surface relief patterns) that can be etched into fused silica. Phase mask fabrication of Bragg gratings, however, is limited in the freedom of the Bragg wavelength, because only one periodicity can be realized. Although this can be overcome by using magnification lenses or by applying strain to a fiber during manufacturing, other techniques allow for more flexibility. Point-by-point inscription, in contrast, builds up a point of refractive index change at a time, and periodicity is implemented through a translation of the waveguide with respect to the light source [1,15]. This allows for the Bragg wavelength, grating length, and overall spectral response to be easily varied. If ultrashort laser pulses from a near infrared laser are used, nonlinear processes, such as multiphoton ionization, can be activated by the occurrence of very high local energy intensities [1,16,17,18]. Because of the polymer’s low absorbance of wavelengths around 800 nm, these processes can further be realized in deeper subsurface regions of a waveguide that might be covered with an additional layer of cladding [19]. 

In this research paper, we present, to the best of our knowledge, the first Bragg grating at an operating wavelength of around 850 nm, inscribed in a planar EpoCore polymer waveguide with near infrared point-by-point Ti/sapphire femtosecond laser technique. First, we briefly show the fabrication of the waveguide by laser direct lithography and the subsequent laser inscription process of the grating. Thereafter, we describe the physical operating principle and characterize the Bragg sensor’s sensitivity to light polarization, strain, humidity, and temperature in detail.

## 2. Materials and Methods

### 2.1. Waveguide Manufacturing

The introduced optical sensing element principally consists out of a Bragg-grating-integrated polymer light waveguide and its surrounding cladding, as well as a supporting substrate. Since automatized visual structure recognition is applied for the inscription of the Bragg grating, an exposed waveguide is formed in the initial processing step. As single-mode light guiding properties in the desired light transmission band are utilized for a robust state measurement, the dimensional prerequisites of the waveguide are simulated beforehand with a commercial software program (Comsol Multiphysics, Comsol, Göttingen, Germany). Due to the low optical attenuation and the adjustable refractive indices, an epoxy-based photo-patternable material compound (EpoCore/EpoClad, Micro Resist Technology, Berlin, Germany) is selected. First, the surface of a square substrate of the material cyclo-olefin copolymer (COP, Zeonor flexible foil, Microfluidic ChipShop, Jena, Germany), with an edge length of 32 mm and a thickness of 188 µm, is activated by oxygen plasma treatment (Plasma Prep, Gala Instruments, Bad Schwalbach, Germany) for 1 min to improve the adhesion of the photoconductive polymers. Afterwards, a 2.0 µm thick homogeneous EpoClad base layer with a refractive index of 1.5708 is applied by spin-coating. This lower coating is pre-baked at 120 °C for 5 min, then UV flood-exposed at a wavelength of 365 nm, and finally hard-baked at 120 °C for 60 min.

The resulting surface is again exposed to an oxygen plasma for 1 min. In the next step, an EpoCore layer of 2.6 µm with a refractive index of 1.5836 is spin-coated. Utilizing a direct lithography machine (μPG 101, Heidelberg Instruments, Heidelberg, Germany), according to a mask template, the pattern of the waveguide is traced by a UV laser with a wavelength of 375 nm and a power of 2.25 mW. The partially cured EpoCore layer is post-baked at 120 °C for 5 min and the sectors without UV exposure are subsequently removed by means of a developer (mrDev600, Micro Resist Technology, Berlin, Germany). Finally, the remaining structure is hard-baked at 120 °C for 60 min. Thus, the produced rectangular polymer waveguide has a height of 2.6 µm, corresponding to the native layer, and a set width of 4.8 µm. Centrally from one substrate edge, the waveguide leads 10 mm in a straight line to the middle. and then bends in a 90° curve with a radius of 5 mm in the direction of the adjacent edge, which is reached with a further straight section of 10 mm. The total length of the produced waveguide is, therefore, approximately 28 mm. The bent design is chosen in order to suppress the influence of scattered light to the greatest possible extent. 

### 2.2. Point-by-Point Laser Inscription

A femtosecond pulsed laser (Ti/sapphire Tsunami/Spitfire pro, Spectra-Physics, Santa Clara, CA, USA) with a wavelength of 800 nm is utilized to inscribe the Bragg grating in the polymer waveguide by applying the point-by-point method [1]. The center of the waveguide is focused with a three-dimensional computer-controlled translation stage (N-565.260 linear translation stage, Physik Instrumente (PI), Karlsruhe, Germany) and an objective lens with a numerical aperture of 0.4 (LD Plan-Neoflur 20×, Zeiss, Oberkochen, Germany). Starting 1 mm from the edge of the sample, the grating points are processed consecutively over a length of 8 mm with a spacing of 2.174 µm, as approximated in advance for a Bragg wavelength around 850 nm of the 8th grating order. The comparatively high point pitch is chosen to ensure a low level of thermal material damage from the energy input of the laser. Each Bragg grating point is a local refractive index modification and results from a single laser pulse with a pulse energy of 28 nJ (PE9-ES-C, Ophir, Jerusalem, Israel) and a pulse length of 95 fs (Autocorrelator Model 109, Spectra-Physics, Santa Clara, CA, USA). After the inscription of the Bragg grating, the upper coating is applied. An EpoClad layer of 20 µm is thereby spin-coated, which is then pre-baked, flood-exposed, and hard-baked according to the parameters mentioned above.

**Figure 1:**
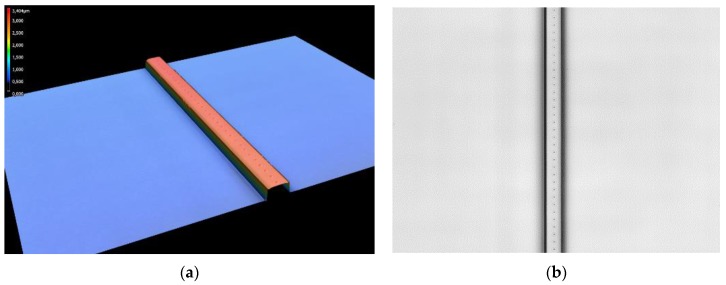
Laser scanning microscope image of the polymer waveguide after point-by-point grating inscription before applying the upper cladding. (**a**) The height profile of the waveguide shows dimensions of 4.841 µm width and 2.572 µm height. (**b**) In the top view, the single refractive index modulated points at a distance of 2.174 µm from each other can be identified.

The central reflection wavelength of the formed polymer Bragg grating is analyzed in the next section. For reliable light coupling and decoupling, it is crucial that both the entry and end facets of the waveguide are planar and free of impurities. Therefore, a 1 mm segment of the sample is first cut off at each edge of the substrate with waveguide ends. Subsequently, the resultant side surfaces are polished. From the coupling point to the beginning of the Bragg grating, the measuring light is guided over a length of approximately 18 mm inside the rectangular waveguide; the decoupling point is located directly at the end of the grating. In Figure 1, two microscopic images of the processed waveguide are shown.

### 2.3. Sensing Principle and Post Processing Characterization

In this section, the fundamental equations for Bragg sensors and the main variables that influence the central Bragg wavelength are presented. In principle, the Bragg grating is a periodic variation of the refractive index in the core of a waveguide leading to a narrow reflection spectrum, whose central wavelength is primarily sensitive to changes in temperature and strain. As shown in the previous section, these modulations are inscribed by the femtosecond laser pulses for each grating point individually, with a grating distance of approximately 2.174 µm. The Bragg condition for the Bragg grating resonance is expressed by Equation (1).

λ_B_ = 2n_eff_Λ/k (1)

Hereby, λ_B_ is the center of the back-reflected Bragg wavelength, n_eff_ is the effective refractive index, Λ is the distance of the single grating points, and k is the grating order (k = 1, 2, 3, ...). Both the effective refractive index and grating period are defined during laser inscription and depend on the temperature and applied strain. If temperature and strain variations from a given starting point occur, the shift in the reflected wavelength Δλ_B_ can be calculated by the total differential of Equation (1), as shown in Equation (2).

Δλ_B_ = 2[Λ(∂n_eff_/∂l) + n_eff_(∂Λ/∂l)] Δl + 2[Λ(∂n_eff_/∂T) + n_eff_(∂Λ/∂T)] ΔT (2)

The left term of Equation (2) represents the effect of the strain and summarizes the change in grating spacing Λ and the induced change of the refractive index. The right term in Equation (2) indicates the effect of the temperature and summarizes the thermal expansion of the material that results in a change of spacing, as well as the refractive index. 

If only mechanical strain is applied to the grating, the thermal term of Equation (2) equals zero and the mechanical term can be simplified, as shown in Equation (3).

Δλ_B_ = λ_B_(1 − p_e_)ε_z_ (3)

The shift in the back-reflected wavelength is linearly dependent on the relative lengthening ε_z_ of the grating in the direction of the waveguide—the center Bragg wavelength in the unloaded condition. Furthermore, p_e_ expresses the effective strain-optic constant. 

If only temperature variations and no mechanical strains occur, Equation (2) can be simplified and expressed as Equation (4).

Δλ_B_ = λ_B_(α_Λ_ + α_n_) ΔT (4)

The shift of the Bragg wavelength is linearly dependent on the temperature change and the center Bragg wavelength under no load condition at a given reference temperature. Here, α_Λ_ expresses the coefficient of thermal expansion (CTE) of the waveguide’s material and α_n_ is the thermo-optic coefficient. 

**Figure 2:**
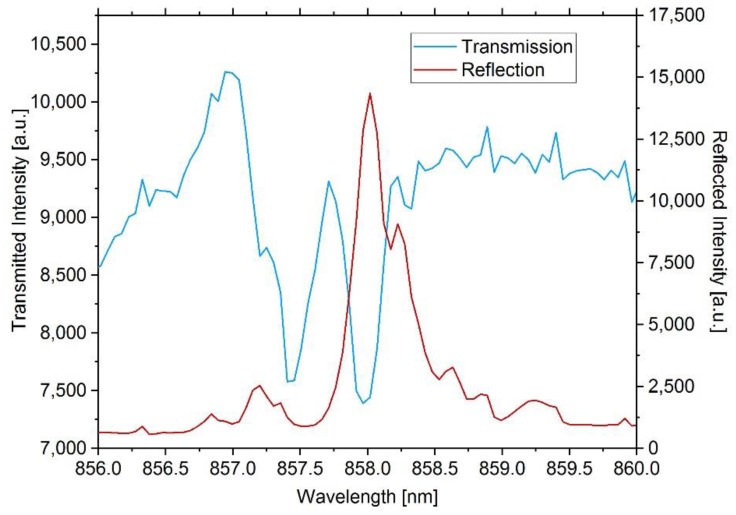
Transmission spectrum of the Bragg grating in free space optical arrangement and reflection spectrum after fiber coupling. The grating of the inscribed sensor reflects approximately 22% of the light coupled into the polymer waveguide. The right shoulder of the reflected Bragg peak probably results from variations of the refractive index modulations of the inscribed grating points and the grating period due to which two narrow Bragg resonance wavelengths can be seen. Two clear dips in the transmitted spectrum indicate losses due to other modes.

After inscription of the grating points, spin-coating of the upper cladding layer, and cutting and polishing of the edges of the sensor element, the integrated Bragg grating is evaluated for the first time. To achieve this, light of a superluminescent diode (SLED) is coupled into a single-mode fiber that is positioned directly in front of the polymer waveguide’s input and another fiber that guides the light from the sensor plate for evaluation into a spectrometer, which is positioned at the output. The transmitted spectrum is shown in Figure 2. Subsequently, the fiber is glued to the waveguide’s input and a reflection spectrum is recorded, which is also depicted in Figure 2.

As can be seen in Figure 2, the transmitted spectrum shows two dips, but only one reflection peak, indicating that more modes than the fundamental mode exist. Based on simulation results (BeamPROP™, Synopsis, Mountain View, CA, USA), higher order modes are in principal conceivable at a wavelength of around 850 nm. However, these are expected to show resonance in the range of several nanometers below the reflected Bragg wavelength. Since no more dips than the two identifiable in Figure 2 can be found in the transmission spectrum, we think that the Bragg grating couples light to a cladding or radiation mode, as reported by Erdogan [20]. Following the calculations for the offset in resonance wavelength due to cladding mode coupling, as reported by Grenier et al. [21], we tried to assign the lower wavelength dip at 857.5 nm to an effective refractive index for any of the possible modes at the Bragg wavelength of 858 nm, but none matched the dips distance of approximately 537 pm. Additionally, the transmission characteristics of similar waveguides without a Bragg grating do not show any conspicuous features that could explain the first dip. However, the occurrence of resonance dips in the transmission spectrum is one specific feature of the femtosecond laser process [22], but theoretical examination remains difficult, as the exact material interaction for EpoCore is not fully understood at present and a detailed study of these matters is beyond the scope of this paper.

Regardless of the specific transmission spectrum characteristic, the back-reflected peak shows sufficient signal-to-noise ratio for an optical state measurement. The behavior under various environmental circumstances is evaluated in detail in Section 3.

### 2.4. Interrogation System

For the permanent evaluation of the Bragg sensor, an in-house-developed interrogation system, whose key properties will be explained in this section, is used. Basically, the interrogator consists of a light source (SLED) (EXS210037-01, Exalos, Schlieren, Switzerland) and a spectrometer (Exemplar, B&W Tek, Newark, DE, USA), which are both connected via a 50:50 coupler. The light emitted by the SLED is proportionally split by the coupler and is guided to the Bragg sensor in a single-mode fiber, from where it is partly reflected, as explained in Section 2.3. The reflected spectrum is again split on its way through the coupler and enters the spectrometer where it is evaluated. The spectrometer sends its data to a personal computer for further processing by software. Additionally to the principle components mentioned, an optical fiber switch (eol 1 × 16 NIR I (near infrared range I (meaning an operating wavelength range from 600–850 nm)), Leoni, Föritztal, Germany) is installed between the coupler and the Bragg sensor that allows the SLED light to be guided to 16 different outputs. Moreover, the key advantage of the interrogation system is its ability to measure the reflected Bragg spectrum independently from the light’s state of polarization (SOP). Light polarization can affect the Bragg wavelength to varying degrees, depending on the particular conditions of the waveguide and the grating itself. To overcome these issues, the SOP of the SLED light coupled into the fiber is randomly changed with high frequency. To achieve this, a free space fiber bench is positioned between the SLED and the coupler, which has two hollow shaft motors in its optical axis that turn with a sufficiently high rotational speed. The motors themselves are equipped with a half-wave (hwp) (FBR-AH2, Thorlabs, Newton, MA, USA) and a quarter-wave retardation plate (qwp) (FBG-AQ2, Thorlabs, Newton, MA, USA), respectively. Because the two motors revolve with different speeds ($v_{hwp}\gg v_{qwp}$), this results in a fast and random variation of the light’s SOP [23] and consequently to different Bragg spectra back-reflected from the sensor. Since the spectrometer is able to capture and transmit more than 900 spectra per second, simple averaging of the data in the evaluation software can be carried out (>100 scans), thus quantifying the central Bragg wavelength without disturbance from the light polarization. Afterwards, the averaged spectrum is used in the software and a Gauss fit for each existent peak is performed. Finally, the mean value for the center wavelength of the fitting result is displayed and stored with a frequency of 1 Hz. The interrogator described here is used to evaluate the Bragg sensor sensitivity to variations of the strain, temperature, and humidity. 

## 3. Results

**Figure 3:**
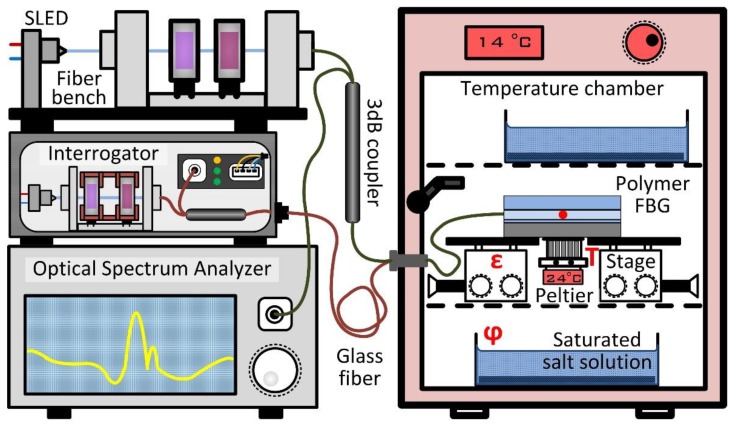
Schematic experimental setup of the investigations carried out for this work. For the polarization study, the light of the superluminescent diode (SLED) is manually readjusted in the fiber bench and guided via the coupler to the sample located inside the temperature chamber. The back reflection is evaluated using the optical spectrum analyzer (OSA). For strain characterization the interrogator with the internal light source, a depolarization unit, coupler, and spectrometer are utilized, and additionally three spectra are captured with the OSA. The sample is positioned on two translation stages, with which the tensile force is applied. The interrogator is also used for temperature measurement, where the sample is located on the Peltier element inside the temperature chamber. To characterize the sensitivity to humidity, various saturated salt solutions are placed in the temperature cabinet. The sample is again evaluated with the interrogator. Note FBG = fiber Bragg gratings.

In this chapter, the investigations for the characterization of the manufactured sensor element are presented individually. By means of Figure 3, the respective experimental setup can be reconstructed.

### 3.1. Polarization Sensitivity

**Figure 4:**
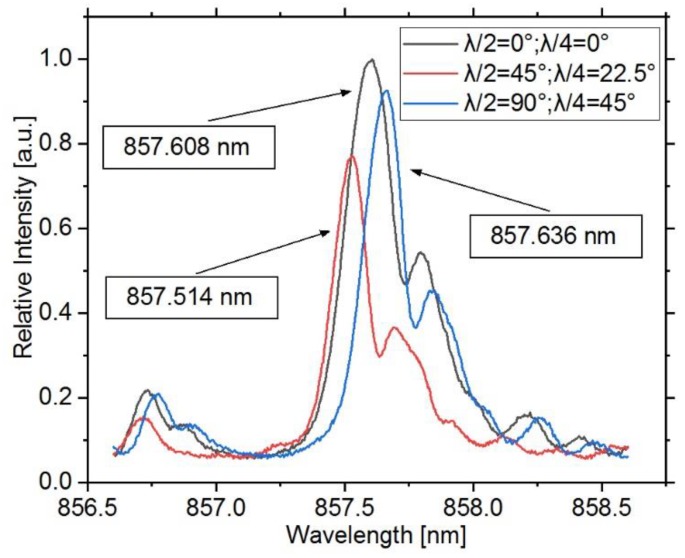
Reflection spectra for the two polarization states, which show the maximum wavelength difference of the main peak. The centers were calculated using Gauss fitting algorithms and differ by 122 pm. Additionally, the spectrum for the linear polarized starting condition is shown.

The influence of the polarization is of particular interest, since it strongly affects the overall accuracy of the measurement. Therefore, the Bragg grating’s reflected spectra in dependence of various SOP is recorded in order to calculate a maximum possible error. Hence, the light of the broadband SLED is again guided via a free space optical setup (fiber bench), in which the retardation plates vary the polarization of the SLED light. According to the manufacturer, the SLED light is linearly polarized with a general polarization extinction ration of 10 to 20 dB (TE/TM), and the degree of polarization is quantified to be 48% at the end of the SLED’s single mode fiber. In order to have a known SOP at the beginning of the optical path, a linear polarizer is put in front of the retardation plates. First the half-wave achromatic plate, and subsequently the quarter-wave plate, change the SOP from linear to any desired polarization states. After the fiber bench, the light is split by means of a 50:50 coupler with one of its outputs connected to the Bragg grating sensor plate. The reflected part of the spectrum is again split in the coupler and guided to an optical spectrum analyzer (OSA) (AQ6373B, Yokogawa, Tokyo, Japan). The spectra are stored for the subsequent evaluation. The retardation plates are gradually rotated by hand (in steps of 22.5°) and the resulting Bragg spectra are captured. In Figure 4, the two spectra with the maximum difference of the central Bragg wavelength after performing a Gauss fit are shown. Additionally, the spectrum for the linear polarized starting condition is plotted. The reader should note that no other disturbance than the alteration of the SOP is likely to occur for this experiment, which is conducted in short time under constant climatic conditions. 

The effective refractive index is a result of the refractive indices of the waveguide, cladding. and grating points, as well as the geometrical dimensions of the waveguide and the positions of the grating points within. The reader should note that it is only valid for a certain light mode, which in turn can be described by its mutually orthogonal polarizations states. Therefore, the Bragg wavelength, as introduced by Equation (1), is in general more or less polarization-dependent, especially for a nonsymmetrical waveguide. In our case, the reflected Bragg wavelengths show a maximum deviation of 122 pm over all recorded polarization states, which is beyond the range of strongly polarization-dependient Bragg gratings in silica fibers. Based on simulation results (BeamPROP™), the difference of the effective refractive indices for both polarization modes is in the order of 5.4 × 10^−5^, and therefore a wavelength difference of approximately 30 pm can be expected, resulting from the waveguide’s asymmetrical geometry. The residual deviation of 92 pm, in contrast, must be a result of the grating point inscription process. The spectrum with the lowest wavelength of 857.514 nm can be assigned to the transverse magnetic mode (TM), while the spectrum with the highest wavelength at 857.636 nm can be assigned to the transverse electric mode (TE). Furthermore, the full width at half maximum (FWHM) of the peaks varies for the different SOP, indicating that the peak with the maximum intensity for the linear polarized starting condition is a superposition of the peaks for both polarization modes. For the peak at 857.514 nm, the light’s SOP after the fiber bench is elliptically polarized, while for the peak at 857.636 nm, the SOP is circular. Between both states, the half-wave plate is turned by 45°, and therefore the light therefore turned by 90°. However, on the way to the grating, the light’s SOP certainly changes due to birefringence, what has to be taken into account for practical usage. 

Therefore, the subsequent results are obtained with the interrogation system presented in Section 2.4 to exclude the potential error originating from different polarization states. For further information on the cause and effect of the SOP, as well as its possible use for sensor applications, valuable studies can be found in the literature [24,25,26]. Furthermore, a table with all recorded values is included in the Appendix A (Table A1). 

### 3.2. Strain Sensitivity

**Figure 5:**
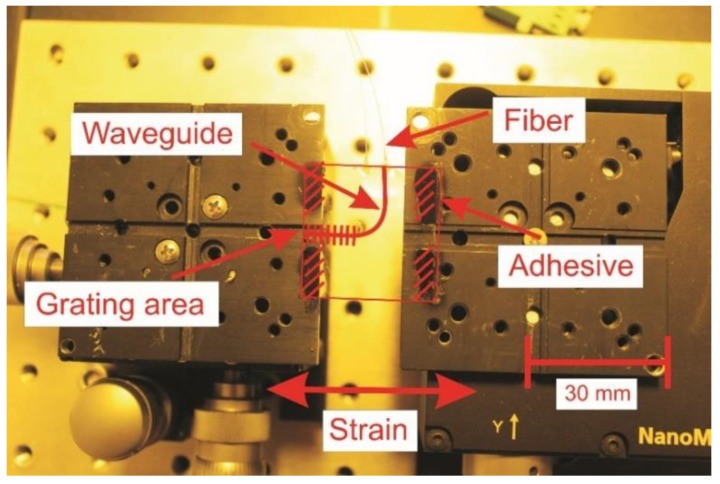
Experimental setup for the strain sensitivity investigation. The dimensions of the sensor plate are 30 × 30 mm. The strain is applied in the longitudinal direction of the Bragg grating in ten incremental steps of 3 µm each. The substrate of the sensor is fixed at its outer edges to the translational stages.

As explained in Section 2.3, a certain strain applied to a Bragg sensor results in a change of the grating point distance, and accordingly in a change of the reflected center wavelength. To investigate this behavior independently from other effects, the sensor is fixed to a stretching setup, as depicted in Figure 5. Two translational stages are positioned at a distance of 18 mm from each other and the sample is fixed to the platforms at the two outer edges. Afterwards, the fine adjustment screw of the right translational stage is utilized to lengthen the sample in the longitudinal direction in 3 µm increments. The total relative elongation is calculated using Equation (5).

ε_z_ = Δl/L (5)

The relative lengthening is expressed in microstrain, and therefore Δl is set in micrometers, while the total length L without strain is given in meters. The abovementioned dimensional changes in the conducted strain experiment result in a maximum relative strain of 1666 µm/m. 

**Figure 6:**
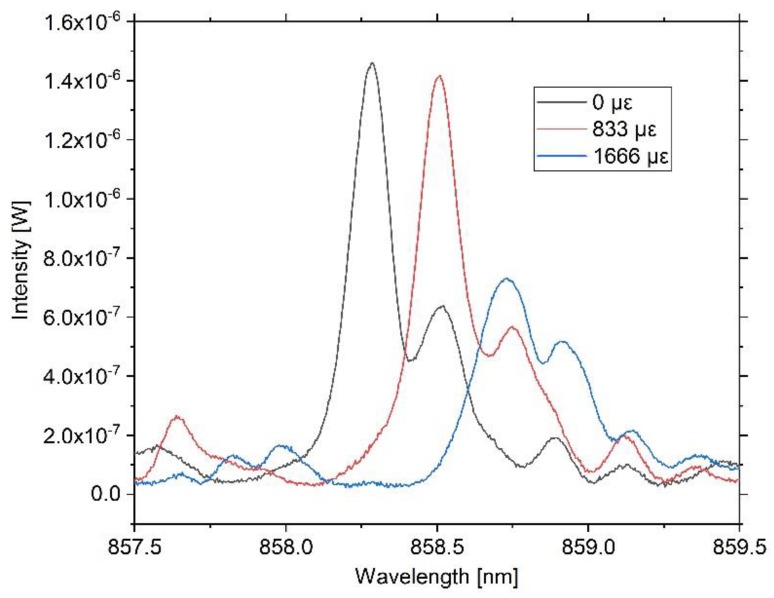
Reflection spectra for three strain conditions. Without applying external strain to the Bragg grating, the center wavelength is 858.285 nm, while for 833 µε the center is 858.509 nm and for a strain of 1666 µε is 858.729 nm. Although in the high strain condition higher attenuation occurs, the peak is evaluated well by the interrogation system. The three spectra are captured with the OSA for qualitative evaluation.

For each step, the reflected wavelength is recorded for 20 s and the sample is stretched and released repetitively in a short-time period, during which the room temperature and relative air humidity are constant at 21 °C and 50%. In Figure 6, three spectra for their associated strain conditions are shown. 

**Figure 7:**
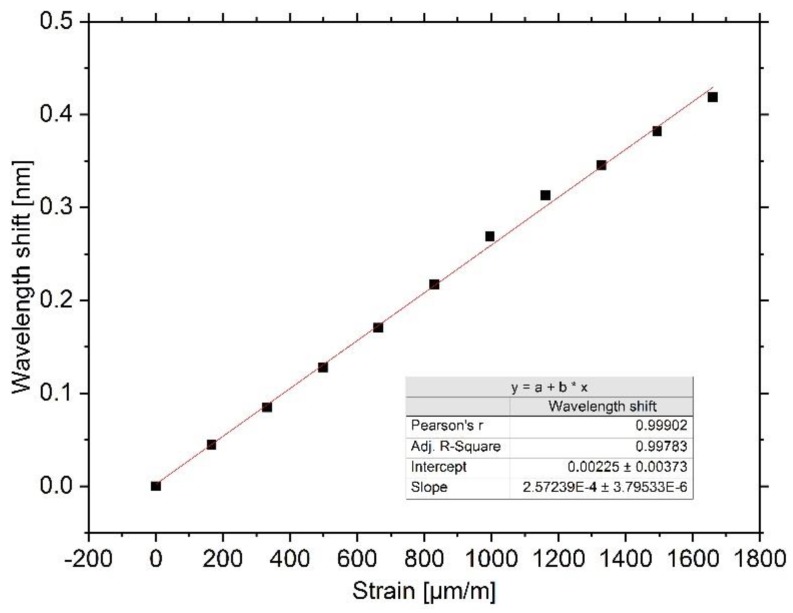
Result of the strain sensitivity experiment. For the investigated range, a linear redshift of the peak with a sensitivity of 0.26 pm/µε exists.

The three spectra shown in Figure 6 represent the starting condition without strain with 833 µε and under maximum load with 1666 µε, respectively. For all three strain conditions, a linear shift of the main peak to higher wavelengths is observable, although for the maximum strain condition an attenuation of the intensity occurs. It is noticeable that for the maximum strain condition, the main peak is attenuated to a greater extent in contrast to the side peak. We believe this substantiates the assumption that the Bragg condition is fulfilled for two adjacent wavelengths, whereby the higher wavelength peak expunges the lower wavelength peak due to scattering effects. Additionally, an overall intensity loss for both peaks can be seen that likely results from dimensional changes of the waveguide, essentially of its height. Nevertheless, due to the superposition of several different effects, it is difficult to evaluate individual processes with certainty and quantify them. As the effect is reversible for the strain range investigated in this experiment, and as the main peak under the high strain condition still shows an adequate intensity, mean values of the center Bragg wavelengths for each strain condition are calculated, which are shown in Figure 7.

For a total lengthening of 30 µm, the Bragg sensor shows linear characteristics. While the wavelength shift for small strains of up to 833 µε is highly linear, the data points for strains greater than 833 µε show a slight deviation from the first order polynomial fitting line. We assume this is caused by the interrogation system’s performance of the Gaussian fitting, which is affected by the occurring attenuation of the spectra, as shown in Figure 6. Nevertheless, with good consistency, the strain sensitivity can be determined to be 0.26 pm/µε. Furthermore, the maximum relative deviation due to hysteresis after stretching the sensor over the full range is 3.76% (16 pm).

However, the obtained sensitivity is less than expected and less than values reported by other groups. Missinne et al. recently reported that their polymeric Bragg sensors have strain sensitivities in the range of 0.85–1.41 pm/µε. They further postulate that the photoelastic constant of optical polymers might be dependent on the Bragg wavelength, since the sensitivity did not scale solely with wavelength for their sensors with different grating pitches [10]. Nevertheless, simple strain variation of the Bragg sensor does not allow precise determination of the photoelastic constant, and further work has to be done. Regardless of the precise value for the sensor in this work, good linear and reversible responses to strain variations are achieved, and further discussion on the strain sensitivity is carried out in Section 4.

### 3.3. Influence of Humidity

To investigate the influence of the relative air humidity on the Bragg wavelength, the sensor is put into an almost hermetically closed, temperature-regulated chamber with a total inner volume of 48 dm^3^. The temperature is set to 14.5 °C and kept constant for the whole time of the experiments. To regulate and stabilize humidity at certain levels, two glass containers with a combined open top surface of approximately 250 cm^2^, containing a saturated salt solution, are put in the upper and lower section of the test chamber. For a “low” humidity level, a saturated solution of magnesium chloride MgCl_2_ is used; for a “mid” humidity level, potassium carbonate K_2_CO_3_ is used; and for a “high” humidity level, sodium chloride is used; resulting in relative air humidity levels of 37.0%, 42.5%, and 62.0%, respectively, after sufficiently waiting for acclimatization. The environmental values are constantly recorded with a digital climate sensor (HYT 939, Innovative Technology, Ebnat-Kappel, Switzerland). The sensor is placed in proximity to the Bragg grating in the middle of the test chamber. The Bragg sensor is evaluated by the interrogation system from outside the chamber, and both are connected by a single-mode glass fiber.

**Figure 8:**
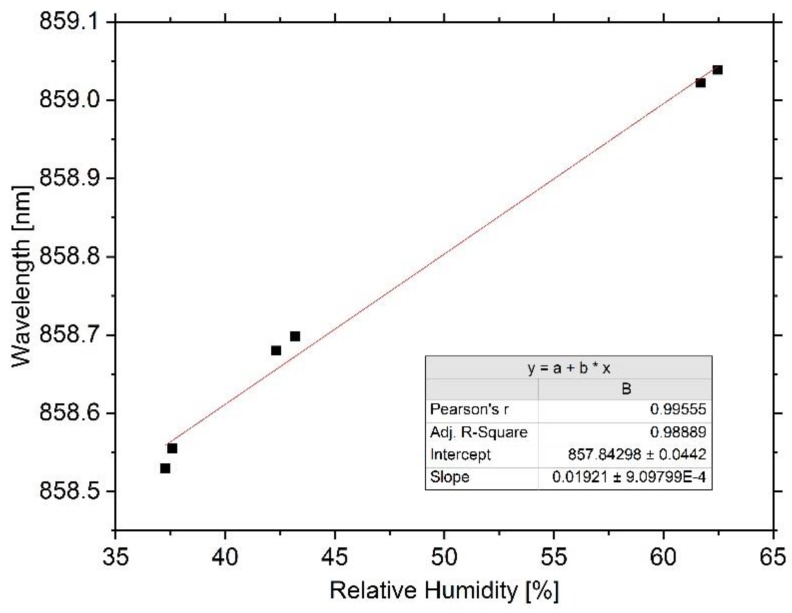
Results of the humidity sensitivity experiment. A linear redshift can be approximated over the investigated range from 37.0% to 62.0%. The sensitivity due to moist air is 19.2 pm/%.

Bragg wavelength, temperature, and relative humidity are captured over multiple days and for all humidity levels. For periods of sufficiently low variations of the inner chamber’s relative humidity, mean values for the reflected Bragg wavelength are calculated, as well as for the corresponding relative humidity; the results re depicted in Figure 8. Due to regular weather changes, the moisture level inside the test chamber is still slightly influenced. Nevertheless, two reference points are identified for each level using the recorded data. 

For the investigated humidity range from 37.0% to 62.0%, a sensitivity of 19.2 pm/% is determined. Under the prerequisite of ensuring sufficient time constants, a linear sensor behavior between the data points can be assumed. However, a generally linear behavior is not assured, since some polymers show nonlinearities [12]. 

Basically, the shift in resonance wavelength is caused by water absorption of the sensor system, due to which volumetric expansion occurs. This can either result from the substrate or the core or cladding polymers. The used cyclo-olefin copolymer substrate is well known for its low water absorption properties (<0.01%), and Yuan et al. report on cyclo-olefin copolymer-based, microstructured polymer optical fiber Bragg gratings with a humidity sensitivity of only 0.26 pm/% [27]. In contrast, Bragg gratings in PMMA usually show higher humidity sensitivities of approximately 40 pm/% [6]. Few data are available on the water absorption properties of EpoCore and EpoClad, however, Gijsenberg et al. proved that the internal stress of EpoClad is significantly reduced due to water absorption [28]. Schmid et al. determined the one-dimensional elongation of SU-8 (a negative epoxy-based photoresist with characteristics similar to those of EpoCore) to be linear, reversible, and with a hygroscopic expansion coefficient of 25.3 ppm/% [29]. Assuming the redshift with increasing humidity is exclusively caused by the volumetric expansion of the EpoCore and EpoClad layers due to water absorption and under consideration of a strain sensitivity of 0.26 pm/µε, this would imply a hygroscopic expansion coefficient of 73 ppm/% for our sensor. However, Schmid et al. also showed that the water absorption characteristics of SU-8 depend strongly on the processing parameters, first and foremost the hard-bake time and temperature. 

### 3.4. Influence of Temperature

**Figure 9:**
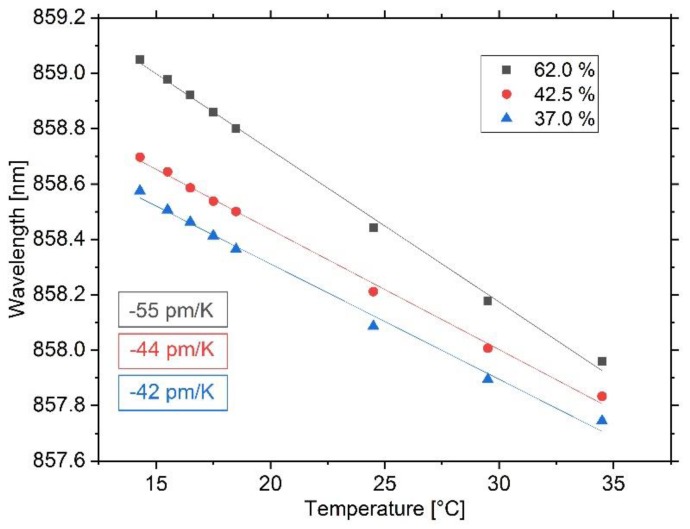
Result of the temperature sensitivity experiment for the three humidity levels. The Bragg grating is heated locally, while the surrounding environment maintains a constant temperature and humidity. At all humidity levels, the sensor shows a linear blueshift, but with increasing sensitivity for higher relative air humidity.

The analysis of the temperature sensitivity of the polymeric Bragg grating is conducted at the same humidity levels that prevail in the previous section. At each level, the sensor plate is put directly on top of a thin metal surface that is temperature-controlled by a Peltier element. Starting again at an environmental temperature of 14.5 °C, the heating plate’s temperature is elevated abruptly to 15.5, 16.5, 17.5, 18.5, 24.5, 29.5, and 34.5 °C, respectively, and the Bragg wavelength is recorded permanently. For each temperature step, after a time of approximately 2 h, a quasi-static condition with a wavelength variation of less than 0.01 pm/s is reached, consecutively mean values for the Bragg wavelength are calculated, and afterwards the Peltier controller is set to idle mode so that the sensor plate reaches its initial equilibrium state. The result is shown in Figure 9.

As stated earlier, polymer optical temperature sensing is of particular interest because of generally larger sensitivities and the opposite sign resulting from a negative thermo-optic constant [5]. For the quasi-static condition of the test environment, a decrease in Bragg wavelength with increasing local temperature is observed. A first order polynomial fit is performed and all curves show linear behavior starting at different wavelengths because of their different moisture contents (see previous section). For the humidity level of 37.0%, the sensitivity is determined to be −42 pm/K, for 42.5% it is −44 pm/K, and for 62% it is −55 pm/K. Furthermore, we calculated the maximum relative deviations due to hysteresis effects to be 2.92%, 1.70%, and 1.20% for the individual relative humidity levels. All of the aforementioned deviations are observed for the last temperature step of 20 K.

However, it is obvious that the decrease of Bragg wavelength does not only result from the thermo-optic constant and the coefficient of thermal expansion, since an increased temperature sensitivity can be seen with increasing environmental humidity. This leads to the assumption that the shift in back-reflected wavelength is a superposition of aforementioned thermal and humidity effects, due to which additional shrinkage occurs. For our sensor, the thermo-optic coefficient of the EpoCore is −71 ppm/K [13], but no manufacturer data about the coefficient of thermal expansion of EpoCore and EpoClad exist, and rare values from literature differ strongly. While Wouters and Puers approximated the CTE of EpoClad to be in the range of 80 ± 10 ppm/K [30], Shi calculated the CTE of EpoCore to be 20–30 ppm/K [31], but obtained this result from a sensor on a glass substrate. Nevertheless, we assume that the dominant thermal expansion is caused by the COP substrate with a CTE of 60 ppm/K, thus we would expect a temperature sensitivity of −11 ppm/K. The residual increase in temperature sensitivity is, therefore, caused by the moisture content of the sensor, mainly its cladding layer, which, for the steady state condition, depends on the environment’s relative humidity.

## 4. Discussion

The Bragg grating inscription by means of near infrared femtosecond laser irradiation using the point-by-point technique shows well-defined refractive index modulations for the utilized polymer material EpoCore. As known for other polymers such as PMMA, this presumably results from high local intensities in the laser focus point enabling nonlinear multiphoton processes [19]. However, the exact increase in refractive index of the grating points has to be verified in future work in order to understand the underlying processes in detail. Nonetheless, the implemented periodic modulation in the waveguide core results in an evident Bragg reflection peak, with sufficient reflectivity for interrogation. In For nonsymmetrical waveguides, special attention on the influence of the SOP has to be attributed. We found the maximum deviation under constant environmental conditions due to variation of the SOP for our Bragg sensor to be 122 pm. This is not only a result of the geometrical waveguide dimensions, but is also influenced by the grating points themselves. To exclude this source of error and to obtain precise measurements, appropriate measures should be taken, for instance by rapidly varying the SOP and recording of averaged values. We focused on the quasi-static sensitivity with respect to temperature, strain, and humidity, and found our femtosecond laser-inscribed Bragg grating to perform similarly to those reported for other polymers and grating inscription techniques [6,10,32]. The sensitivity to strain is 0.26 pm/µε, sensitivity to temperature is −44 pm/K (at 43% relative humidity), and sensitivity to humidity is 19 pm/%, although these values are still influenced by the utilized substrate and cannot satisfy the requirement of general validity. Additionally, we calculated the maximum error due to hysteresis to be 3.76% in the case of strain variations and to be 2.92% in the case of temperature variations. For the variation of the relative humidity, no hysteresis could be determined because of superimposed transverse influences due to control inaccuracies. Besides, the specified sensitivity to humidity suffers from restricted significance, as a larger range of measuring points is required for a general statement. The investigation of the influence of the relative air humidity will be addressed in future work, as the sensor’s moisture content seems to dominate the overall sensing characteristics. 

**Figure A1:**
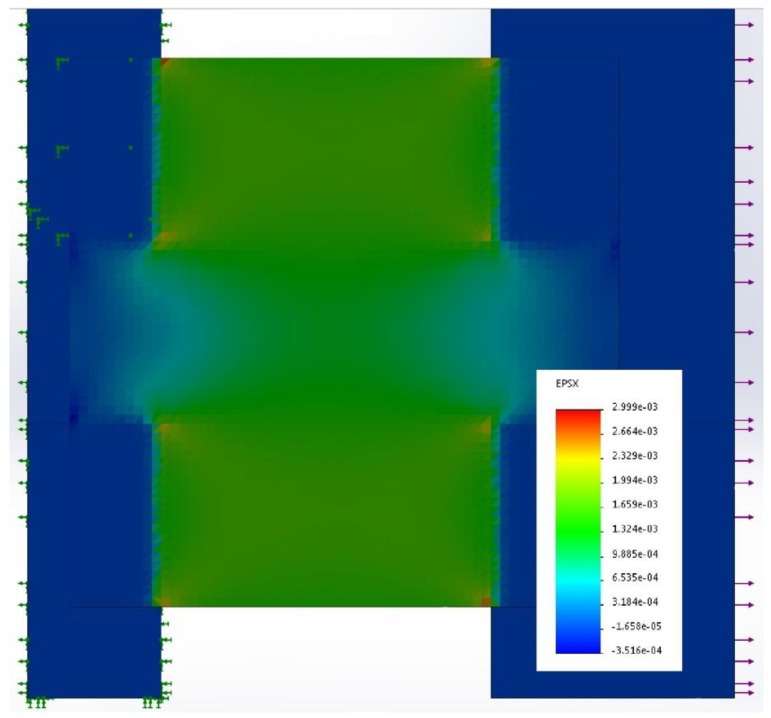
Top view of the result of a static strain analysis for the polymeric sensor element with SolidWorks 2018. In the simulation, the left stage is set to be fixed (indicated by the green arrows), while the right is defined as a slide bearing. A force of 28 N is applied to the right stage (purple arrows), thus resulting in a total lengthening of 30 µm between the two stages. The result shows the strain in the sensor plane in the direction of the force. Because the substrate is fixed at its outer edges to the stages by means of adhesive, the applied force results in a lower strain in the sensor area, as could be expected from the total lengthening under the assumption of uniform strain distribution. Qualitatively, the simulation result shows a strain approximately 2–3 times lower in the sensor area (color blue to green) compared to the values in the area between two fixed points (green).

For the design of a polymer Bragg sensor system, the choice of substrate regarding thermal and mechanical properties has to be carefully considered. In the conducted strain experiment, a comparatively low sensitivity to strain was determined. We assume that this characteristic originates from anisotropic strains within the polymer. As can be seen in Figure 5, the substrate is fixed at its outer corners to the stages, and therefore the applied force predominately results in larger material stress in the outer areas compared to the grating area. The reader should note that the incremental lengthening of 3 µm is measured globally between the translational stages, thus the effective strain and the resulting stress within the waveguide may not be specified in general terms. A simple computer-aided mechanical stress analysis proved the uniaxial loading results in the aforementioned strain distribution (see Figure A1 in the Appendix B). Although uncertainty for the exact sensitivity remains, in measuring operation, the sensor shows good linear and reversible response to strain variations.

However, it is remarkable that the moisture content of the sample plays a crucial role for the sensitivity, as shown for the different temperature sensitivities for different levels of relative humidity. Girschikofsky et al. found the sensitivity to relative humidity of their Bragg gratings to be around −8 pm/% for a sensor manufactured with the phase mask technique and operating at a wavelength of 1530 nm in OrmoCore^©^, a hybrid polymer that is known for its lower water absorption in contrast to epoxy-based photoresists [12]. Nevertheless, the sensor did not have a cladding layer, and was therefore more affected by a reduction of the effective refractive index instead of swelling due to water absorption. Hence, we assume that unwanted humidity cross-sensitivity could be overcome, not only by choosing proper polymers with low water absorption, but also by adjusting the cladding height. Unfortunately, few data are currently available. Considering this, specific work on the water absorption of optical photoresists should be carried out in order to determine the polymer Bragg sensors best suited for specific measuring tasks.

A distinct feature of the utilized point-by-point inscription with femtosecond laser pulses in photoconductive polymer waveguides is the easy manufacturing of nonstandardized grating structures to enable the processing of sensors in a flexible manner. Future work will focus on better understanding of the underlying processes during inscription of the Bragg grating and the transfer to different substrates and light-guiding polymers, alongside determination of the linearity and working range for practical usability.

In the long term, we aim to demonstrate the suitability of the obtained foil-integrated sensor on the example of status monitoring of a lithium ion cell in combination with a recently developed polymeric arrayed waveguide grating interrogator [33] to realize an all-polymeric sensing system. Finally, we can state that for a time scale of six months, no degradation of the femtosecond laser-inscribed sensor is observable. During all experiments carried out in this work, the sensor showed reasonable repeatability and high robustness.

## Appendix A

**Table A1:** Recorded values for the polarization experiment. The wave plates’ relative positions are relative to the linear polarizer in front of them.

Wave Plates Relative Positions (Half; Quarter)	Delta Main Peak	Delta Main Peak	Delta Side Peak	Delta Side Peak
[deg]	[pm]	[%]	[pm]	[%]
0; 0	0	100	0	100
0; 22.5	10.72	106.14	9.76	94.66
0; 45	5.44	87.97	−4.48	77.73
0; 77.5	4.16	85.83	−10.72	80.58
0; 90	−17.12	97.05	−16.96	95.00
22.5; 0	−86.40	102.33	−35.20	81.28
22.5; 22.5	−79.68	108.31	−25.44	98.35
22.5; 45	−56.96	105.10	−23.68	94.36
22.5; 77.5	−46.24	91.30	−29.92	79.85
22.5; 90	−15.52	99.75	−20.16	84.11
45; 0	−88.80	72.95	−50.40	56.00
45; 22.5	−94.08	77.20	−124.64	67.46
45; 45	−87.36	89.33	−62.88	73.77
45; 77.5	−84.64	89.66	−69.12	70.53
45; 90	−77.92	68.48	−51.36	52.67
77.5; 0	0.80	86.50	−17.60	74.40
77.5; 22.5	−16.48	59.78	−27.84	56.35
77.5; 45	−65.76	64.90	−30.08	61.15
77.5; 77.5	−59.04	85.37	−36.32	75.09
77.5; 90	−64.32	91.87	−38.56	70.81
90; 0	−25.60	100.54	−16.80	99.86
90; 22.5	21.12	107.70	−7.04	96.12
90; 45	27.84	92.84	−9.28	83.31
90; 77.5	10.56	93.42	−3.52	91.75
90; 90	−10.72	109.21	−1.76	99.06
0; 0	−1.49	102.58	−2.16	94.23
